# Dementia in the developing world

**DOI:** 10.1590/S1980-57642009DN30100001

**Published:** 2009

**Authors:** Ricardo Nitrini

**Affiliations:** Editor-in-Chief

Cognitive impairment and dementia are often studied as if the social and economical features of the region or country in which they occur have no bearing on their characteristics. Numerous studies performed across different regions have shown the prevalence of dementia to increase at a similar rate and Alzheimer’s disease to be the predominant cause of dementia in almost all countries when the same methods and criteria for the diagnosis have been adopted thus lending credence to this notion of independency of cognitive disorders from socioeconomic background and even from ethnic features of the populations under study.

However, there are several regional differences that warrant special attention from investigators, medical teams and public health authorities. First of all, the diagnosis of cognitive disorders is based largely on performance in tests that are themselves influenced by cultural characteristics of the population. The instruments used for diagnosis in developing countries were often originally devised for use in a developed and culturally different country. Along these lines, education plays a significant role in the diagnosis of cognitive impairment and dementia. In populations with low or heterogeneous educational level, reaching a diagnosis is much more complex than in more educated and homogeneous populations. Neuropsychologists in developing regions are particularly concerned with these differences.

Risk factors for dementia such as arterial hypertension, diabetes mellitus, dyslipidemia and infectious diseases have different prevalence among different regions. Furthermore, adequate treatment of these conditions by the primary care health system, as well as the treatment of the patients with dementia, is heavily dependent on the regional economic situation.

Since its beginnings, many articles have been published by **Dementia & Neuropsychologia** on the influence of cultural characteristics or low educational attainment on performance in neuropsychological tests, and this issue carries articles which revisit this theme.

Moreover, in this issue we are publishing two papers on a condition that deserves special mention: Chagas disease. This disease has a high prevalence in Latin America where it is well known by the disorders it causes in the cardiac and gastrointestinal systems. The central nervous system involvement in this disease, although described by Carlos Chagas, is not yet well understood.

The editorial board of Dementia & Neuropsychologia considers that publishing papers on conditions that cause cognitive impairment in developing regions and which are not highly prevalent in the developed world, is one of the main motivations for the existence of the journal. There are many diseases that share this profile and in forthcoming issues we shall be publishing reviews on these disorders. The main focus is to highlight the significance of these conditions in a bid to improve knowledge and to encourage researchers to prioritize them as an important field for research.

**Ricardo Nitrini** Editor-in-Chief

